# Design and Synthesis of Novel Peptides to Protect Ferulic Acid against Ultraviolet Radiation Based on Domain Site IIA of Bovine Serum Albumin

**DOI:** 10.3390/biom11091285

**Published:** 2021-08-27

**Authors:** Yinghan Wu, Hisham N. Farrag, Tamaki Kato, Hua Li, Shinya Ikeno

**Affiliations:** 1 School of Food Science and Engineering, Yangzhou University, 196 Huayang West Road, Yangzhou 225127, China; wu.yinghan277@mail.kyutech.jp (Y.W.); muziwanghua@163.com (H.L.); 2 Department of Biological Functions Engineering, Kyushu Institute of Technology, Graduate School of Life Science and Systems Engineering, Kitakyushu Science and Research Park, Kitakyushu 8080196, Fukuoka, Japan; farrag.hisham320@mail.kyutech.jp (H.N.F.); tmkato@life.kyutech.ac.jp (T.K.)

**Keywords:** ferulic acid, UVA light, cyclic peptide, bovine serum albumin

## Abstract

Ferulic acid (FA) is known for its excellent antioxidant properties, which can provide many health benefits. One of its drawbacks is its instability under UVA light, which limits its potency. In this study, the new peptides LW2 (QNKRFYFRKNQ) and CW2 (a cyclic form of LW2) were designed based on bovine serum albumin site IIA conformation. A UVA irradiation experiment was performed to investigate the protective ability of these peptides towards FA against UVA damage. The percentages of FA remaining under UV irradiation due to the protection of CW2 and LW2 were 83% and 76%, respectively. The results showed the importance of the cationic residues and hydrophobic residues included in the peptide sequences. Moreover, the cyclic rigid structure showed greater protective ability as compared to its linear counterpart.

## 1. Introduction

Ferulic acid (4-hydroxy-3-methoxycinnamic acid, FA) is a type of phenolic compound which exists in fruits, vegetables, and grains in a free form or bonded to the cell wall [1]. FA has an excellent antioxidant capacity, with many physiological properties including anti-inflammatory, antioxidant, antimicrobial [2], anticancer [3], and antidiabetic activity [4]. Therefore, it has a wide range of applications as a food supplement, in many pharmaceutical formulations [5], and in the cosmetics industry [6]. Nowadays, people are taking greater care of their health and have become aware of the use of antioxidants such as FA in their food regimen [5,7].

However, its applications are limited because of its unfavorable physicochemical properties, which include autoxidation [8], poor water solubility, and light sensitivity. FA is extremely sensitive to ultraviolet (UV) rays, which is naturally exists as a part of sunlight. It has been reported that when FA is exposed to UVA, photo-oxidation occurs and reactive oxygen species (ROS) are formed [9]. Those reactions may destroy the hydroxyl group attached to the benzene ring and the alkene group included in the FA structure [10]. Thus, these drawbacks should be taken into consideration during the processes of formulation, storage, and transportation. To solve these problems, a suitable protective excipient for FA is considered an ideal strategy. Many materials have been investigated as appropriate agents for such purposes, for example hydroxypropyl-β-cyclodextrin [11], solid lipid nanoparticles [12], and gelatin [4].

**Figure 1:**
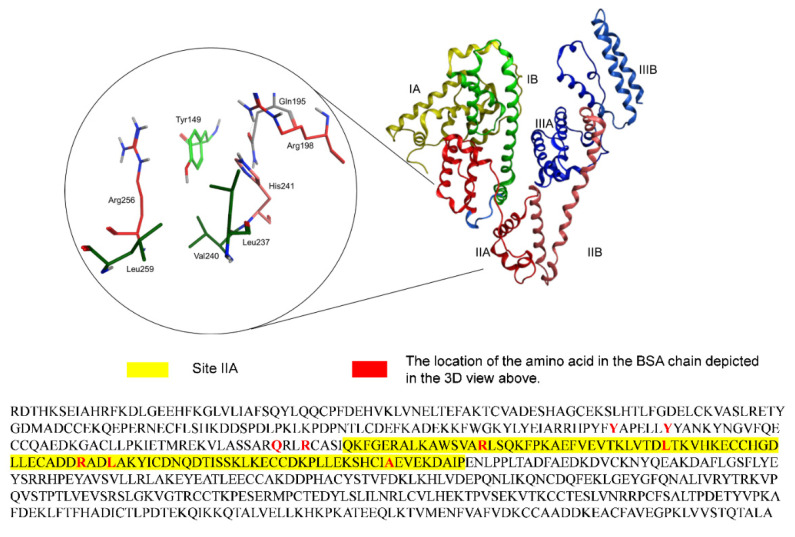
Domain structure of BSA site IIA and the full amino acid sequence of BSA. The heart-shaped BSA consists of 3 homologous α-helical domains (I, II, III), and each domain contains 2 subdomains (A and B).

However, using the above materials complexed with FA to form a stable complex, over-consumer reagents and long-term stirring are required. Proteins, which are major biomolecules, have a variety of functions; one of these is a protective function. In a sense, proteins can be said to be mild materials with protective functions because they are environmentally safe, and their structure naturally contains certain sub-domains that allow target molecules to conveniently bind or be covered. Bovine serum albumin (BSA) is often used as a protective protein, and its applications are diverse in the protection of macroscopic and microscopic molecules. BSA is well regarded as a standard model protein in vivo carrier, and its active binding site has already been studied [13]. It has reported that there is a high possibility of FA binding to BSA at subdomain site IIA of BSA due to the interaction forces including hydrogen bonds, hydrophobic interactions, van der Waals forces, and electrostatic forces. As shown in Figure 1, the subdomain IIA structure of BSA, including Leu 237, Tyr 149, Arg 256, Glu 152, Ser 191, Tyr 156, Glu 291, Ala 290, Arg 198, Arg 217, and His 241, shaped a hydrophobic pocket. In a previous report, the main interaction force between BSA and FA was a hydrogen bond by Arg 256 [14], and the hydrophobic amino acids strengthened these interactions by hydrophobic interaction forces [15]. Interestingly, the damage of FA caused by UVA decreased after BSA was added to the solution. In other words, BSA offered some protective support to FA against UVA in the solution. There is no doubt that BSA is an excellent carrier material, but as BSA is formed of macromolecules and FA is composed of small molecules, using BSA to protect FA is overkill.

The general term “peptides” refers to molecules consisting of short chains of amino acids connected by amide bonds, and many peptides with various functions have been reported [16]. Among them, peptides with protective functions have been reported [17], some protecting against protein aggregation and others protecting small molecules [18]. However, peptides in general have a linear structure and are easily degraded by proteases. On the other hand, cyclic peptides are expected to stabilize the steric structure and improve stability against enzymatic degradation. In this study, we designed the novel cyclic peptide CW2 based on the structure of BSA site IIA and evaluated the protective function of FA from UVA.

To mimic the BSA site IIA structure, QNKRFYFRKNQ (CW2 or LW2) was engineered with 4 cationic residues (2 Arg and 2 Lys), 4 polar residues (2 Asn and 2 Gln), and 3 hydrophobic amino acids (2 Phe and Tyr). Two Arg residues were added to facilitate hydrogen bonding between peptides and FA. Simultaneously, two more Lys residues were applied to improve the electrostatic interaction. Furthermore, three hydrophobic amino acids, Phe-Tyr-Phe, were used to mimic BSA’s hydrophobic field. Since CW2 and LW2 are both cationic peptides, we created an anionic cyclic peptide CEG (EGWGEG) for comparison.

## 2. Materials and Methods

### 2.1. Materials

The calculated masses of CW2, protected LW2, deprotected LW2, and CEG were computationally determined as 1509.795, 3256.576, 1528.743, and 615.229, respectively, using ChemDraw 20.0 software. The preparation of linear peptide followed the standard solid-phase peptide synthesis method (SPPS) using 9-fluorenylmethoxycarbonyl (Fmoc) chemistry and 2-chlorotrityl resin as the solid support [19]. All Fmoc-protected amino acids, resin, piperidine, *O*-benzotriazole-*N*,*N*,*N*′,*N*′-tetramethyluronium hexafluorophosphate (HBTU), 1-hydroxybenzotriazole hydrate (HOBt·H_2_O), *N*,*N*-diisopropylethylamine (DIEA), 1-ethyl-3-(3-dimethylaminopropyl) carbodiimide hydrochloride (EDC·HCl), and 2,2,2-trifluoroacetic acid (TFA) were purchased from Watanabe Chemical Industries, Ltd. (Hiroshima, Japan). Ferulic acid and other reagents were purchased from Wako Pure Chemical Industries, Ltd. (Osaka, Japan).

### 2.2. Synthetic Procedure

**Scheme 1:**
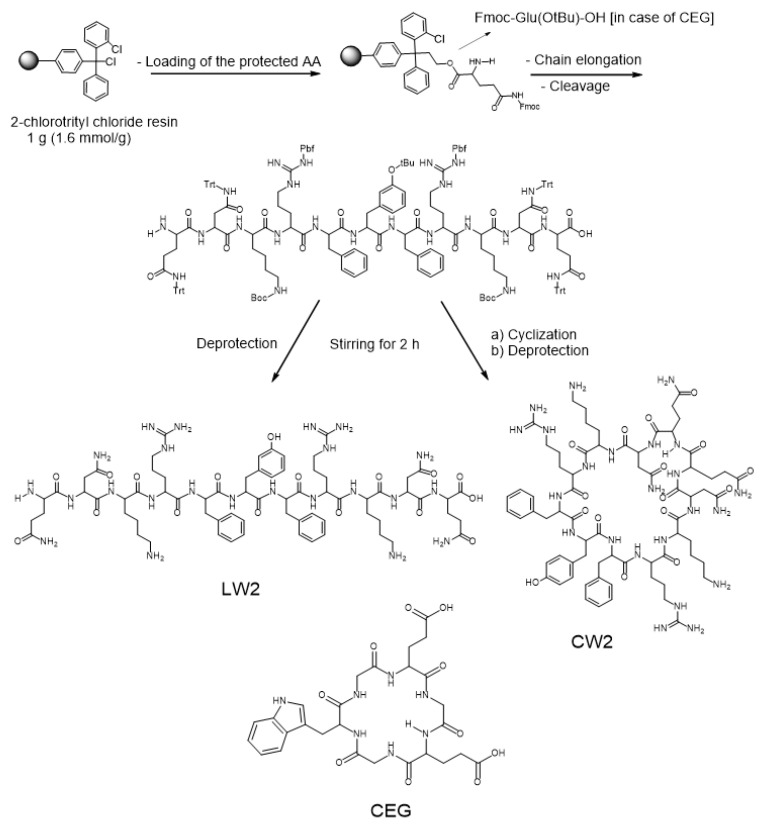
SPPS synthetic protocol of the LW2, CW2, and CEG peptides.

Standard SPPS was used to synthesize the peptides LW2, CW2, and CEG (Scheme 1). The synthetic process started with the loading of 1 mmol Fmoc-glutamine amino acid onto 1.6 mmol 2-chlorotrityl chloride resin, the addition of 0.7 mL DIEA in 30 mL dichloromethane (DCM) for reaction, and rotation for 1 h (the entire reaction was performed at room temperature, as below). After adding 1 mL methanol to cover the unreacted sites on the resin, the reaction was continued by rotating for 30 min. The following sidechain-protected amino acids were coupled to the loaded glutamine residue using 2 mmol HBTU, 0.7 mL of DIEA, and 2 mmol of HOBt in 30 mL of N, N-dimethylformamide (DMF) as a solvent for the coupling process. The coupling process was carried out over 2 hours. The Fmoc-protecting groups were removed by 20% (*v*/*v*) piperidine in DMF (30 mL), which was accomplished in 30 min. The cleavage of the sidechain-protected linear undecapeptide from the resin was performed using a mixture solution including 2,2,2-trifluoroethanol (TFE) (18 mL)/acetic acid (6 mL)/dichloromethane (DCM) (6 mL) at a ratio of 3:1:1 (*v*/*v*/*v*). The removal of the sidechain-protected group was carried out over 2 hours. Purification was carried out by using a semi-preparative RP-HPLC Hitachi L-7100 apparatus fortified with a Chromolith^®^ performance RP-18e (4.6 × 100 mm, Merck, Germany). The mobile phases were acetonitrile containing 0.1% TFA (solvent B), and H_2_O containing 0.1% TFA (solvent A). The elution gradient was 0–100% of solvent B in 30 min. The flow rate was adjusted to 1.300 mL/min at room temperature. The peak intensity was determined both at a wavelength of 220 nm. The removal of the sidechain-protected groups was achieved using a mixture of TFA/trisisopropylsilane (TIS) at a ratio of 99:1 (*v*/*v*). Lyophilization was carried out in an FD-1000 freeze dryer (Tokyo Rikakikai, Japan). A cyclization reaction was carried out following a low concentration (0.5 mM) of LW2 which efficiently avoids dimer formation. Then, 2 equiv. of EDCI·HCl, and 4 equiv. DIEA were used for the cyclization process. The removal of the sidechain-protected groups of CW2 was carried out as mentioned before. Purification was achieved by a semi-preparative RP-HPLC followed by lyophilization to yield the targeted pure final cyclic product [16]. CEG synthesized follow the same process. The synthetic process was elucidated by HPLC as shown in Supplementary Figures S1–S3. All of the synthesized peptides were confirmed by fast atom bombardment (FAB^+^), and time-of-flight (TOF) mass spectrometry, and the masses were found as follows: LW2 (sidechain-protected), LW2, CW2, and CEG values were 3259.600 [M + 3H]^+^, 1528.812 [M + H]^+^, 1509.400 [M]^+^, and 638.217 [M + Na]^+^, respectively, as depicted in Supplementary Figure S4.

### 2.3. Preparation of Protein–FA Complex Solution

The stock solution of FA was prepared in MOPS (3-(*N*-morpholino) propanesulfonic acid, 20 mM, pH 7.0, 0.1% TFA) buffer at a final concentration of 100 µg/mL. CW2, LW2, CEG (tested compounds), and BSA (positive control) were also prepared in MOPS buffer, each at a final concentration of 1 mg/mL. FA (25 µM) was mixed with CW2 (200 µM), LW2 (200 µM), CEG (487 µM), BSA (10 µM) solution; the molar ratios of FA:peptide/BSA were 1:8, 1:8, 1:20, and 1:0.4. The blank control, including 25 µM FA in MOPS, 200 µM CW2/LW2/CEG, and 10 µM BSA, was also prepared.

### 2.4. UVA Irradiation

The tested group (LW2-FA, CW2-FA, CEG-FA), BSA-FA, and peptide/protein-only groups were irradiated with UVA using a UVA lamp (365 nm, UVL-56 Handheld UV Lamp, 6 W; UVP, USA) at the same time. The distance between the lamp and the samples was fixed at 20 cm. All the samples were measured at 319 nm (UV-vis 1200, Shimadzu, Japan) using 10 mm cuvettes with MOPS buffer as a blank. The contents of FA samples were checked at 0, 2, 4, 8, and 12 h. The reservation ratio (RR) of FA was determined according to the following formula:

RR = {(Abi − Abj)/(Abo − Abj)} × 100%

where Abi is sample absorbance including FA at 319 nm as time, Abj is sample absorbance without FA at 319 nm as time, and Abo is the absorbance of the initial sample at the same wavelength. The UV-visible absorption spectra were scanned at the same time, in the range from 250 to 400 nm. All experiments were carried out in triplicate.

### 2.5. Simulation Analysis

Molecule docking was simulated using Molecular Operating Environment 2018.0 (MOE, Chemical Computing Group, Montreal, QC, Canada) software with MOE operation (2016 version) with simulation of the interaction between FA and BSA/CW2. Briefly, the crystal structure of BSA was acquired from the Protein Data Bank (PDB Code: 3V03), and Chain A of the structure was selected for further simulation. The protein receptor and ligand atoms were prepared using Quick Prep program, minimizing the energy at the force field of default values (Amber 10: EHT). The binding site was selected by the site finder using the alpha center. Default values (Triangle Matcher) were selected for the docking parameters [20].

## 3. Results

### 3.1. The Reservation Ratio of FA under UVA Irradiation

**Figure 2:**
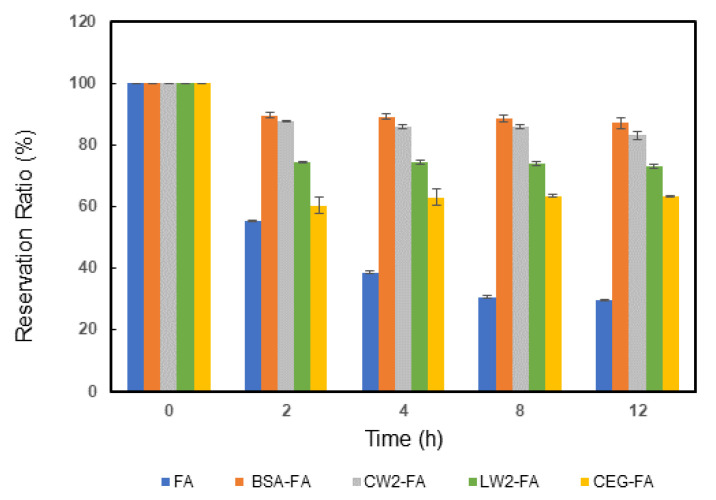
The time dependence of relative FA concentrations under UVA radiation. The concentration of FA was 5 µg/mL in all samples. In addition to the blank control, the other groups included the 0.6 mg/mL BSA, 0.3 mg/mL CW2, 0.3 mg/mL LW2, and 0.3 mg/mL CEG groups. Error bars represent means ± SD (*n* = 3).

The content of FA decreased depending on the exposure time, and the pattern of decrease slowed down steadily (Figure 2). For the blank, the descending portion happened within the first 2 h; its RR was left at only 55.4% after 2 h of exposure. It was shown that almost half of the FA was destroyed in this period. Because FA is unstable under neutral/alkaline conditions, UVA depredates FA easily [14,21]. On maintaining the UVA irradiation up to 12 h later, the solution became stable and its RR value held at 29.6%. In the BSA-FA group the RR improved 2.8 times, and the value was increased from 29.6% to 86.5% compared with the blank control. BSA showed highly effective protection and stability against the damage of FA from UVA. As compared to BSA-FA, the CW2-FA tested group showed similar protection ability, but the RR value was slightly lower than that of the BSA-FA group at 83%. The LW2-FA tested group also had a positive effective result, as the RR was kept at 73%. This signaled the main factors influencing the interaction between peptides and FA, especially cyclic peptides. A cyclic hexapeptide (CEG) was selected. This cyclic peptide included 1 tryptophan, 2 glutamic acids, and 3 glycine residues. In theory, this peptide should be negatively charged in physiological solution, meaning that FA was barely attached to the peptide. In the CEG-FA group, after 12 h of UVA exposure the RR remained at 63%. In summary, the protective effect was as follows: BSA > CW2 > LW2 > CEG.

### 3.2. UV Scanning Spectra

**Figure 3:**
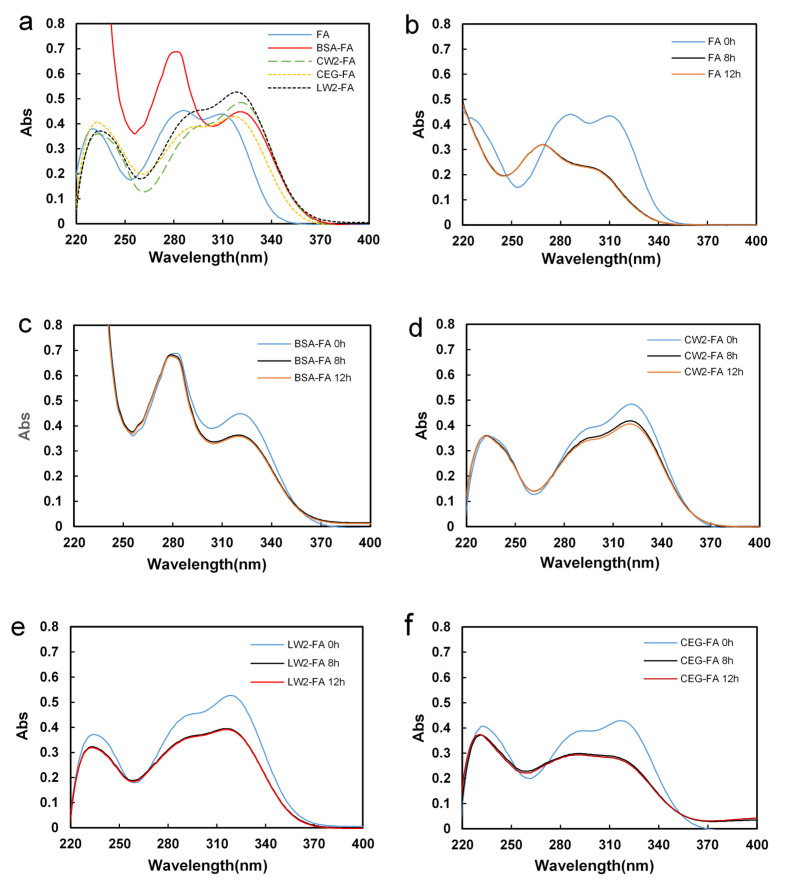
Time dependence of the UV absorption of each sample in the wavelength range of 220 nm to 400 nm (**a**) before UVA irradiation. (**b**) FA, (**c**) BSA-FA, (**d**) CW2-FA, (**e**) LW2-FA, and (**f**) CEG-FA.

Figure 3a shows the UV spectrum of the blank control, BSA-FA, CW2-FA, CEG-FA, and LW2-FA before UVA irradiation. The UV spectrum of FA exhibits 3 λmax peaks at 230 nm, 287 nm, and 310 nm, respectively. In the BSA-FA group, the λmax 310 nm bathochromically shifted 2 nm, and λmax 287 nm was covered since protein has a strong absorption peak at 280 nm [14]. In the CEG-FA group, the 3 λmax peaks were bathochromically shifted 2–3 nm. CW2-FA/LW2-FA showed a similar UV spectrum to CEG-FA, but more bathochromically shifted peaks shifted down and up [6]. That indicated the different mechanisms of interaction between CW2/LW2-FA and BSA/CEG-FA.

Figure 3b shows the blank (FA only) scanning spectrums at different irradiation times. The 287 nm peak disappeared and a new peak was shown at 260 nm. The λmax 310 nm peak had a certain hypsochromic shift with the degree of shift depending on time, and the absorption gradually decreased at the same time.

In the BSA-FA, CW2-FA, and LW2-FA groups, FA was slightly damaged by UVA. After 12 h of exposure (Figure 3c–e), the spectrum was shown to be only slightly degraded, with no bathochromic or hypsochromic shift. This is because damaged FA may exist in the solution in free form, with no attachment to the protein/peptides. However, in the CEG-FA group (Figure 3f), the spectrum showed a similar decreasing process, although to a lesser degree compared with the blank control. CEG showed some protective function, but this was not very efficient.

### 3.3. Concentration Dependence of the Protection of FA

**Figure 4:**
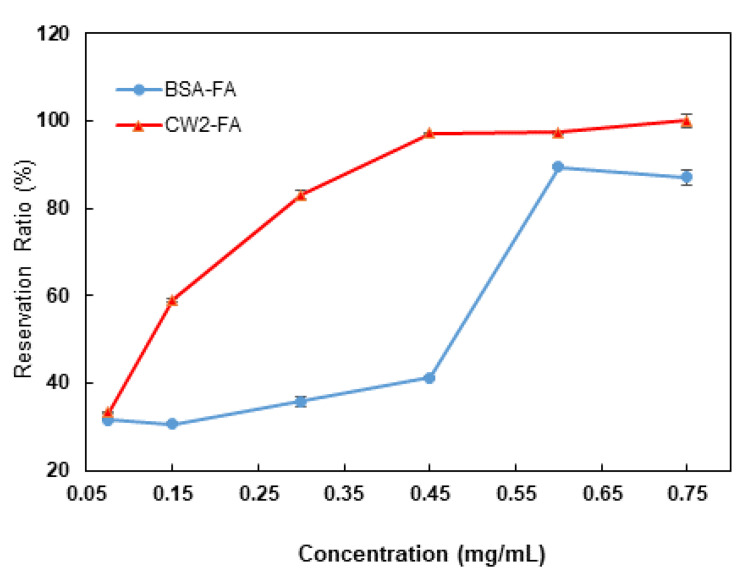
The reservation ratio of FA depending on the dose after exposure for 12 h under UVA. The concentration of FA was 5 µg/mL in the 2 groups. The blue line indicates the reservation ratio of BSA-FA groups after 12 h of UVA irradiation with a series of protein concentrations (0.075 mg/mL, 0.15 mg/mL, 0.3 mg/mL, 0.45 mg/mL, 0.6 mg/mL, and 0.75 mg/mL). The red line indicates the reservation ratio of CW2-FA groups, following the same treatment and concentrations as CW2. Points represent means ± SDs (*n* = 3).

The RR of FA depending on the dose after exposure for 12 h under UVA is shown in Figure 4. CW2-FA and BSA-FA had no protective function for FA against UVA damage at a 0.075 mg/mL concentration of protein or peptide. BSA and CW2 started to show a protective function of FA at 0.45 mg/mL and 0.15 mg/mL, respectively. The dose effect in the CW2-FA group was shown earlier than in the BSA-FA group. As concentrations increased, the protective ability was increased. The increasing curves were slightly different, with the protection of CW2 increasing gradually depending on the dose and that of BSA sharply increasing at a certain concentration. These results show that CW2 effectively protected FA in relation to the amount added.

## 4. Discussion

**Figure 5:**
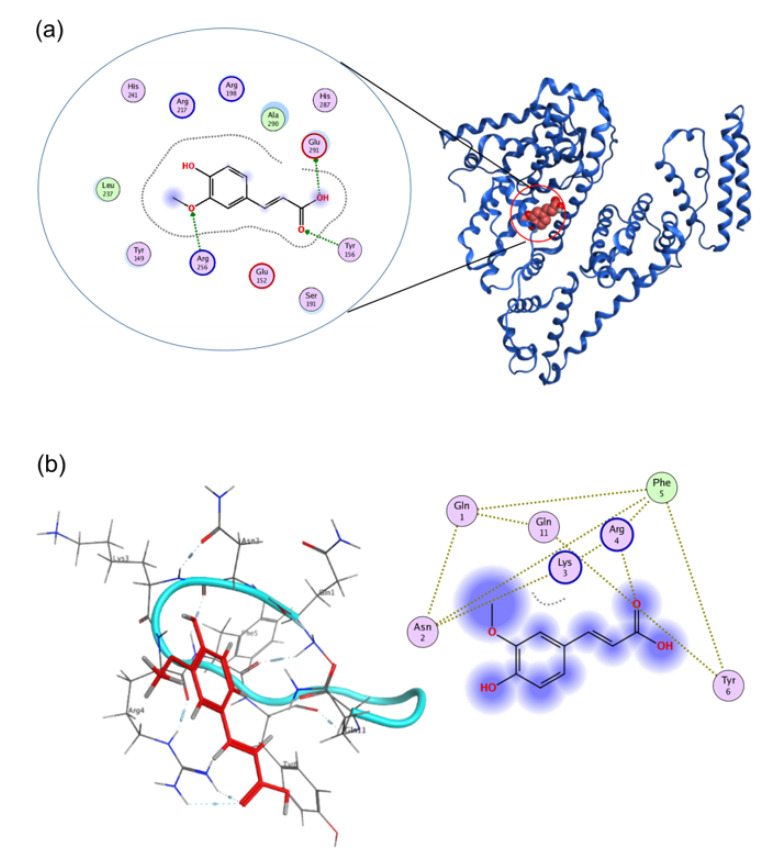
Molecular binding analysis using the Molecular Operating Environment. (**a**) A 3D image of FA docked onto the BSA (PDB: 3V03), and a 2D representation of the binding site and interacting residues between FA and BSA. (**b**) A 3D image of FA docked onto the CW2 and a 2D representation of the binding site and interacting residues between FA and CW2.

The subdomain IIA structure of BSA includes several hydrophobic amino acids (Leu 237, Tyr 149, Tyr 156, and Ala 290) and positively charged amino acid (Arg 256). As previously reported, Arg 256 has a role as a main contributor in the hydrogen bonding between BSA and FA [14], and the hydrophobic amino acids strengthen these interactions by hydrophobic interaction forces, as shown in Figure 5a. CW2 was designed to have activity similar to the BSA binding activity which exists within subdomain IIA [20]. To mimic such functional structures, the new peptides CW2 and its linear counterpart were designed to have 2 Arg, 2 Lys (cationic amino acids), and 1 hydrophobic site containing (Phe-Tyr-Phe) to enhance the connection between peptide and FA in Figure 5b. The structural activity relationship was confirmed by the UVA exposure experiment. The experimental results showed the protective effect of the newly designed peptides towards FA against UVA. The cyclic peptide showed a more efficient protective function than the linear peptide. Therefore, the cyclic structure of CW2 can work as a better protective shield towards FA because of its rigid structure [22]. At the same time, the positively charged residues are also very important for obtaining protective function. That was proven by using CEG, which did not exhibit a good protective effect towards FA against UVA radiation.

## 5. Conclusions

In this study, the new cyclic peptide CW2 was designed and synthesized to imitate BSA activity in the protection of FA against UVA. CW2 and LW2 (the linear counterpart) both showed the ability to protect FA against UVA damage. The RR values were maintained at 83% and 76%, respectively, for a reaction time of 12 h. CW2 showed a higher capacity to keep FA stable than the linear form. The negatively charged cyclic peptide CEG had the lowest protective ability (the RR was kept at 63%) compared to the others. Therefore, positively charged and hydrophobic residues are required to initiate the interactions between FA and peptides. We suggest that the use of peptides to protect phenolic compounds against UVA damage requires the presence of a protective cyclic conformation and cationic and hydrophobic residues. These functional peptides, which can be chemically synthesized to produce uniform molecules, will become useful as protective agents to replace proteins, but cyclic peptides are currently expensive for practical use. Over the years, peptide ligation technologies have been developed that provide useful tools to facilitate successful cyclic peptide synthesis. These include enzyme-mediated cyclization, a cost-effective approach for large-scale synthesis [23]. In the future, it is hoped that such peptide synthesis methods can be improved so that cyclic peptides can be synthesized inexpensively. As a prospect, it will be necessary to conduct cytotoxicity analyses, antibacterial tests, and molecular stability tests for practical applications with the goal of developing FA for food additives and cosmetics for skincare purposes due to their excellent antioxidant properties.

## Data Availability

The authors confirm that the data supporting the findings of this study are available within the article.

## Supplementary Materials

The following are available online at https://www.mdpi.com/article/10.3390/biom11091285/s1. Figure S1: HPLC chart of the sidechain-protected (LW2 and CW2). Figure S2: HPLC trace analysis of the deprotected sidechains (LW2 and CW2). Figure S3: HPLC trace analysis of the pure cyclic deprotected CEG. Figure S4: Mass spectra of peptides. Table S1: The score of molecular docking between BSA/CW2 and FA. Table S2: Yield and purity for synthesized compounds.
