# Supplementary material for: Design and Synthesis of Novel Peptides to Protect Ferulic Acid against Ultraviolet Radiation Based on Domain Site IIA of Bovine Serum Albumin
Source: Biomolecules. 2021 Aug 27;11(9):1285. doi: 10.3390/biom11091285 (PMC8472342; doi:10.3390/biom11091285)
Supplement: Supplementary file 1 [file biomolecules-11-01285-s001.zip › biomolecules-1354734-supplementary.pdf]

Supporting information

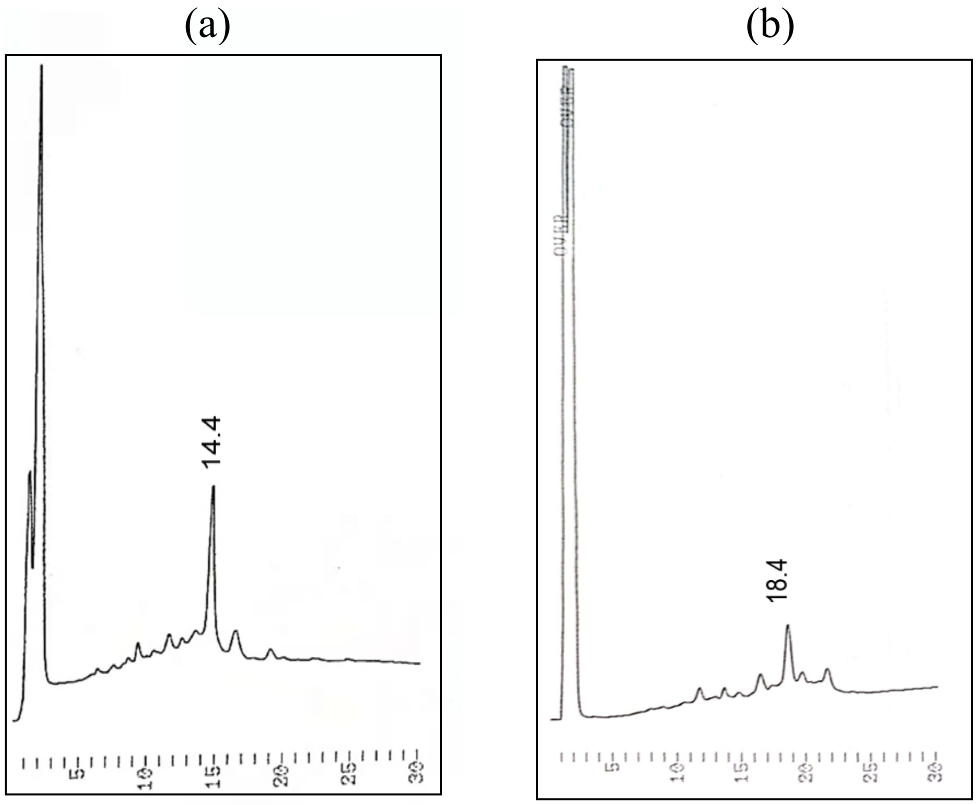

Figure S1 HPLC chart of the protected sidechain [LW2] and [CW2]. Column: Chromolith® performance, RP-18e (4.6 × 100 mm), Flow Rate: 1.300 mL/min, Eluent: 50– 100 % B in 30 min, Solvent A: 0.1% TFA in H_2O Solvent B: 0.1% TFA in CH_3CN, Detect: UV 220 nm. (a) protected sidechain linear [LW2] a retention time, RT= 14.4 min; (b) protected sidechain cyclic [CW2] a retention time, RT=18.4 min.

(a) (b)

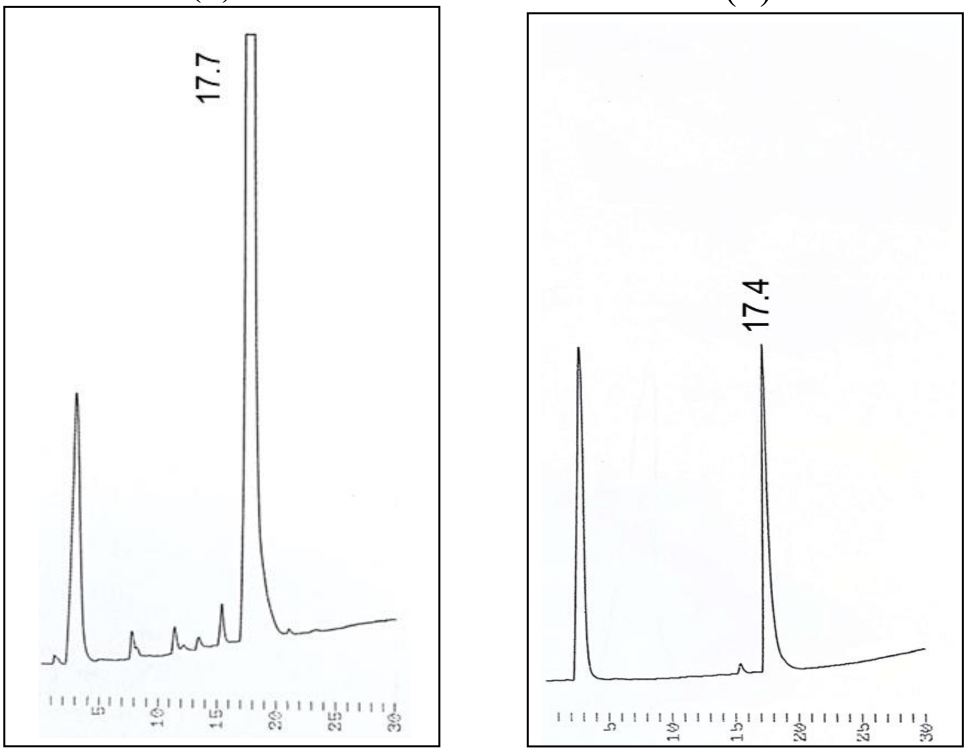

Figure S2 HPLC trace analysis of the deprotected sidechain [LW2] and [CW2]. Column: Chromolith® performance, RP-18e (4.6 × 100 mm), Flow Rate: 1.300 mL/min, Eluent: 0 – 100 % B in 30 min, Solvent A: 0.1% TFA in H_2O Solvent B: 0.1% TFA in CH_3CN, Detect: UV 220 nm. (a) HPLC trace analysis of [LW2] a retention time, RT= 17.7min; (b) HPLC trace analysis of [CW2] a retention time, RT= 17.4min.

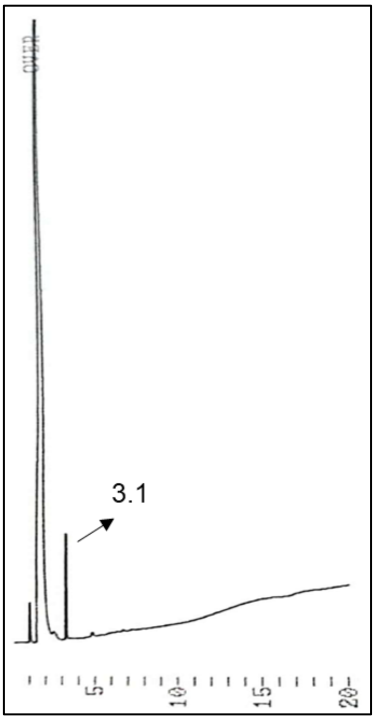

Figure S3 HPLC trace analysis of the pure cyclic deprotected CEG using solvent B gradient of 0-100%. RT= 3.1 min. Column: Chromolith performance, RP-18e (4.6 × 100 mm), Flow Rate: 2.000 mL/min, Eluent: 0 – 100 % B in 15 min, Solvent A: 0.1% TFA in H_2O Solvent B: 0.1% TFA in CH_3CN, Detect: UV 220 nm

Figure S4 Mass spectra of thesis peptides. Identification of the synthesized compound was done by Fast Atom Bombardment (FAB) + mass spectroscopy (a, b, c) and Electrospray Ionization Time-of-Flight (ESI-TOF) mass spectrometry (d) for the determination of the molecular weight of the compound.

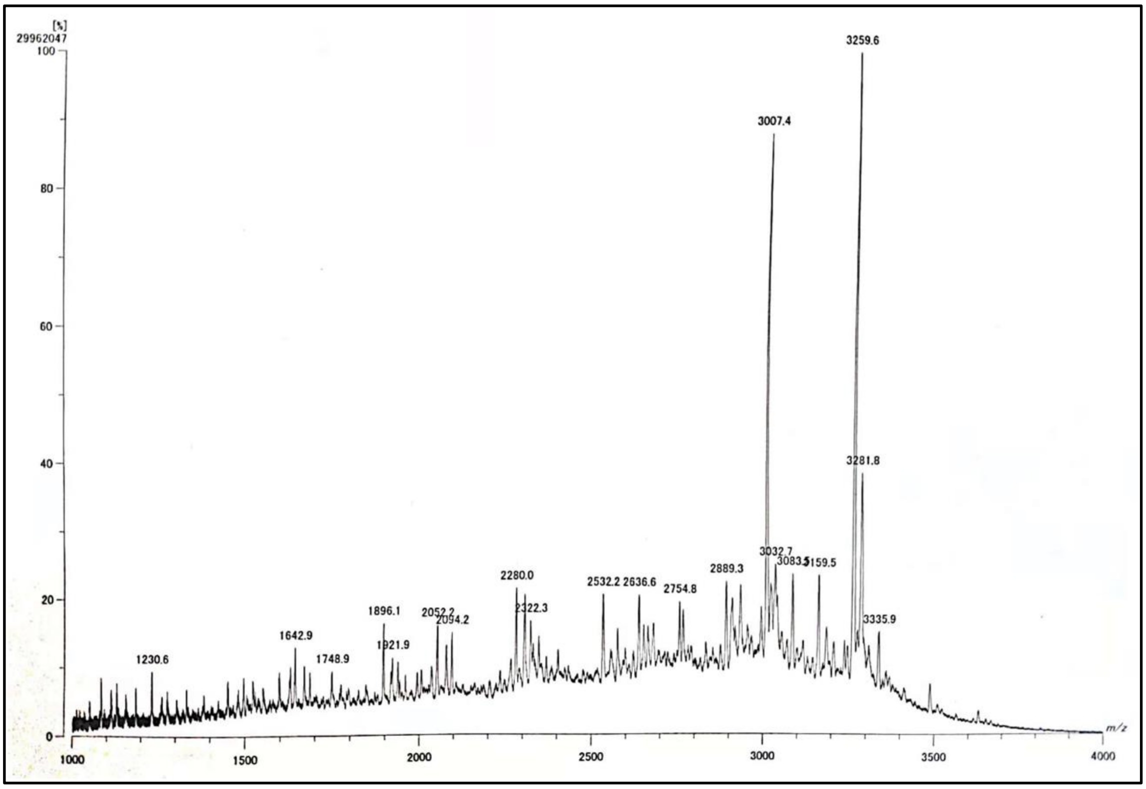

(a) FAB$^+$ Mass spectrum of protected LW2 $[M+3H]^+$

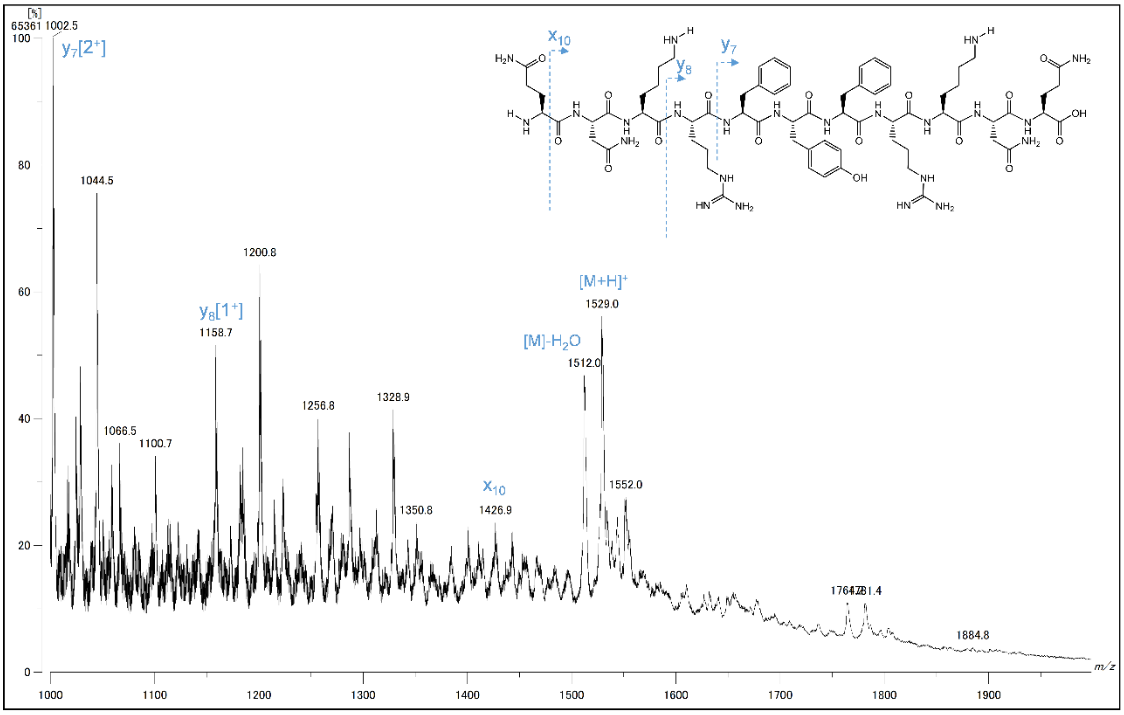

(i)

Inlet : Direct *Ion Mode : FAB+*
RT : 3.26 min *Scan# : 27*
Elements : 12C 69/0, 13C 0/0, H 106/106, N 23/23, O 17/17
Mass Tolerance : 1000ppm, 5mmu if m/z < 5, 50mmu if m/z > 50
Unsaturation (U.S.) : -0.5 – 50.0

	Observed m/z	Int%	Err[ppm / mmu]	U.S.	Composition
1	1528.8122	100.00	-1.0 / -1.5	28.5	12C69 H106 N23 O17

(ii)

(b) FAB$^+$ Mass spectrum of LW2 [M+H]$^+$, (i) FAB$^+$ Mass fragementation pattern of LW2, (ii) FAB$^+$ Mass data of LW2.

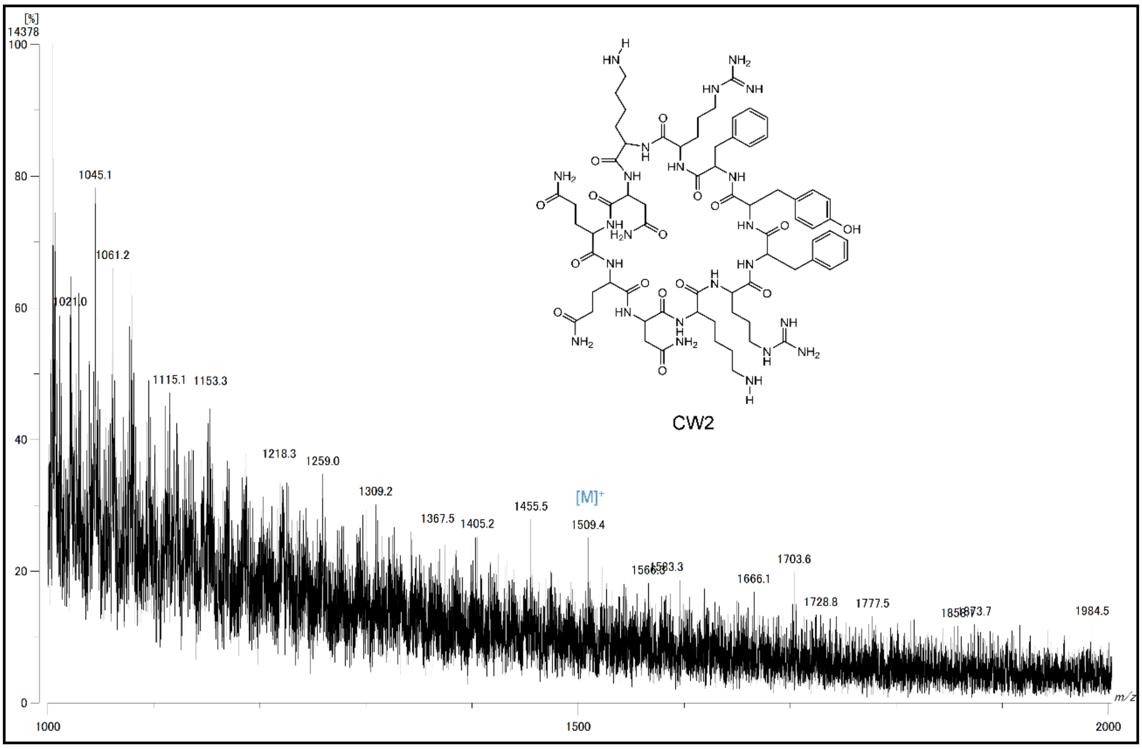

(c)FAB$^+$ Mass spectrum of CW2 [M]$^+$

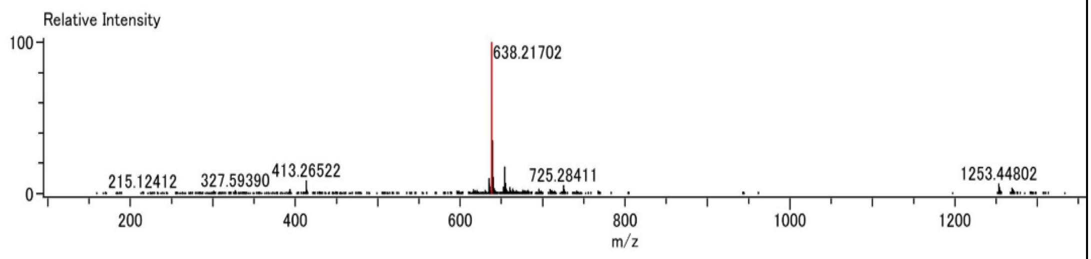

Mass	Intensity	Calc. Mass	Mass Difference [mDa]	Mass Difference [ppm]	Possible Formula	Unsaturation Number
638.21702	190522.56	638.21866	−1.64	−2.57	$^{12}C_{27}{}^{1}H_{33}{}^{14}N_{7}{}^{23}Na_{1}{}^{16}O_{10}$	14.5

(d)ESI-TOF Mass spectrum of CEG $[M+Na]^+$

Table S1 The score of molecule docking between BSA/CW2 and FA.

Name	S-Score	Rmsd-Refine	E-Refine
BSA	-8.3599	1.6010	-24.5977
CW2	-4.2035	1.9949	-16.4607

Table S2 Yield and purity for synthesized compounds.

Peptide Name	Yield (%)	Chromatographic Purity (%)
LW2	97.02	97.4
CW2	50.24	98.3
CEG	45.86	>99.0
